# Targeting mitochondria by lipid-selenium conjugate drug results in malate/fumarate exhaustion and induces mitophagy-mediated necroptosis suppression

**DOI:** 10.7150/ijbs.102424

**Published:** 2024-10-28

**Authors:** Xing Chang, Hao Zhou, Jinlin Hu, Teng Ge, Kunyang He, Ye Chen, Rongjun Zou, Xiaoping Fan

**Affiliations:** 1 Guang'anmen Hospital of Chinese Academy of Traditional Chinese Medicine, Beijing, China.; 2 Senior Department of Cardiology, The Sixth Medical Center of People's Liberation Army General Hospital, Beijing 100048, Beijing, China.; 3 Department of Cardiovascular Surgery, Guangdong Provincial Hospital of Chinese Medicine, the Second Affiliated Hospital of Guangzhou University of Chinese Medicine, the Second Clinical College of Guangzhou University of Chinese Medicine, Guangzhou 510120, Guangdong, China.; 4 Guangdong Provincial Key Laboratory of TCM Emergency Research, Guangzhou 510120, Guangdong, China.; 5 State Key Laboratory of Dampness Syndrome of Chinese Medicine, Guangzhou 510120, Guangdong, China.; 6 Xianning Medical College, Hubei University of Science & Technology, Xianning 437000, China.

**Keywords:** Atherosclerosis, Mitophagy, Mitochondrial, Necroptosis, Endothelial Injury.

## Abstract

Atherosclerosis (AS) is a chronic vascular disease primarily affecting large and medium-sized arteries and involves various complex pathological mechanisms and factors. Previous studies have demonstrated a close association between atherosclerosis and inflammatory damage, metabolic disorders, and gut microbiota. It is also closely linked to several cellular processes, such as endothelial cell pyroptosis, ferroptosis, mitophagy, mitochondrial dynamics, and mitochondrial biogenesis. Mitophagy has been recognized as a previously unexplored mechanism contributing to endothelial injury in atherosclerosis. Our study aims to further elucidate the potential relationship and mechanisms between AS-induced mitophagy dysfunction and the interaction of TMBIM6 and NDUFS4. Data from the study demonstrated that atherosclerosis in AS mice was associated with substantial activation of inflammatory and oxidative stress damage, along with a marked reduction in endothelial mitophagy expression and increased pathological mitochondrial fission, leading to mitochondrial homeostasis disruption. However, under pharmacological intervention, mitophagy levels significantly increased, pathological mitochondrial fission was notably reduced, and oxidative stress and inflammatory damage were suppressed, while necroptotic pathways in endothelial cells were significantly blocked. Interestingly, the deletion of TMBIM6 or NDUFS4 in animal models or cell lines markedly impaired the therapeutic effects of the drug, disrupting its regulation of mitophagy and mitochondrial fission, and leading to the re-emergence of inflammatory responses and oxidative stress damage. Metabolomics analysis further revealed that autophagy plays a pivotal regulatory role during drug intervention and after genetic modification of TMBIM6 and NDUFS4. The activation of autophagy (macroautophagy/mitophagy) alleviated the negative effects of mitochondrial fission and inflammatory damage induced by lipid stress in endothelial cells, a regulatory mechanism likely associated with the TMBIM6-NDUFS4 axis. Subsequent animal gene modification experiments demonstrated that knocking out TMBIM6-NDUFS4 negates the therapeutic effects of the drug on lipid-induced damage and metabolic function. In summary, our research reveals a phenotypic regulatory mechanism of endothelial cell stress damage through mitophagy, influenced by the interaction of TMBIM6 and NDUFS4. Pharmacological intervention can restore mitochondrial homeostasis in endothelial cells by regulating mitophagy via the TMBIM6-NDUFS4 pathway. This novel insight suggests that TMBIM6-NDUFS4 may serve as a key therapeutic target for atherosclerosis.

## Introduction

Atherosclerosis is a critical pathophysiological basis of cardiovascular diseases and can lead to endothelial damage, myocardial infarction, cerebral infarction, peripheral vascular disease, and various other cardiovascular events^1^-^3^. It is one of the leading health threats and a major public health challenge worldwide. Lipid metabolism disorder is the underlying pathological basis of atherosclerosis, characterized by arterial lesions that originate from the intima. The initiating factors often involve lipid and complex carbohydrate accumulation, hemorrhage, thrombus formation, fibrous tissue proliferation, and calcification, leading to plaque formation in the arterial media^2^. As atherosclerotic lesions progress, plaques may develop to the point of obstructing arterial lumens, resulting in ischemia or necrosis in the tissues or organs supplied by the artery, with potentially severe consequences such as acute coronary syndrome and stroke^4^, ^5^. Atherosclerosis, as a chronic inflammatory response, involves structural and functional changes in multiple cell types, such as vascular endothelial cells, smooth muscle cells, and macrophages^6^.

Mitophagy, a specialized form of autophagy, eliminates damaged or malfunctioning mitochondria, thereby preserving cellular homeostasis and ensuring normal function^7^, ^8^. Under the influence of lipotoxicity and inflammatory stimuli, endothelial cells experience mitochondrial depolarization following damage, which subsequently activates mitophagy-related proteins^9^, ^10^. This process involves the recognition of dysfunctional mitochondria caused by disrupted mitochondrial homeostasis, followed by the encapsulation of the damaged mitochondria into mitophagosomes, which then fuse with autolysosomes^11^, ^12^. Within autolysosomes, damaged mitochondria are degraded by lysosomal enzymes. In ubiquitin-dependent mitophagy, the PINK1/Parkin pathway plays a pivotal role. PINK1, a serine/threonine protein kinase, is normally transported to the mitochondrial inner membrane and degraded^13^, ^14^. However, when mitochondrial damage occurs and membrane potential is lost, PINK1 accumulates on the outer membrane of the mitochondria, recruits and activates Parkin through phosphorylation, and ubiquitinates autophagy receptors like Mfn1, Mfn 2, and VDAC1^15^. These ubiquitinated receptor proteins subsequently bind to LC3 on the surface of autophagosomes^16^. The molecules released by mitophagy are recycled for new protein synthesis and energy production^17^, ^18^.

Recent evidence emphasizes the critical role of mitophagy in endothelial injury and cellular metabolic dysfunction^19^. Under physiological or tolerable stress conditions, vascular endothelial cells regulate thrombus formation, vascular inflammation, and vascular tone to maintain vascular homeostasis^20^. Mitophagy is crucial in endothelial injury, contributing to heightened thrombosis, vascular leakage, and inflammation. Further research has shown that the activation of PINK1-driven mitophagy facilitates the removal of mitochondria damaged by copper oxide nanoparticles in endothelial cells, indicating the protective role of mitophagy in endothelial mitochondrial pathway cell death^21^, ^22^. Further research has shown that melatonin can mitigate oxidative stress and enhance endothelial cell function by modulating mitophagy-related pathways. Melatonin upregulates PINK1 and Parkin protein expression, activates LC3, and enhances mitophagy. Experimental studies also revealed that H_2_O_2_-induced oxidative stress significantly activates the transcription and expression of IP3R and VDAC^23^, leading to calcium overload in microvascular endothelial cells. This calcium overload results in the abnormal opening of mitochondrial permeability transition pores (mPTP), the dissipation of mitochondrial membrane potential, and activation of mitochondrial pathway endothelial cell death^22^. However, melatonin intervention can inhibit endothelial calcium overload through MAPK/ERK pathway activation, preserving mitochondrial morphology and structure integrity and preventing endothelial cell apoptosis through mitochondrial pathways. Further studies indicated that the loss of PINK1 or Parkin genes impairs mitophagy activation, leading to increased mitochondrial fragmentation and resulting in mitochondrial dysfunction and metabolic disorders in endothelial cells, with significant programmed cell death (apoptosis, necroptosis, pyroptosis, ferroptosis) observed in these cells.

Necroptosis is a regulated process of endothelial cell death that serves a key role in controlling cell death during lipotoxicity-induced inflammatory storms^24^, ^25^. Necroptosis can be induced by various innate immune signaling pathways, all of which lead to the phosphorylation and activation of RIPK3^26^, ^27^. RIPK3 is required for necroptosis induced by death receptors and activates the pseudokinase mixed-lineage kinase domain-like protein (MLKL), which undergoes conformational changes and localizes to the plasma membrane, causing changes in membrane permeability and regulating programmed cell death^28^, ^29^. Previous studies have shown that RIPK3 activation exacerbates endothelial necroptosis during ischemia-induced endothelial damage, leading to microvascular barrier dysfunction, increased vascular permeability, inflammatory storms, and microvascular spasms. Melatonin pharmacological intervention blocks RIPK3 activation, inhibiting the RIPK3-PGAM5-CypD-mPTP cascade and reducing endothelial necroptosis^30^. Based on the above findings, mitophagy dysfunction may mediate necroptosis through mitochondrial pathways in endothelial cells^31^.

Gynostemma pentaphyllum, a plant from the Cucurbitaceae family, contains Gypenoside A as its primary active compounds^32^, ^33^. Previous studies have found that Gynostemma pentaphyllum, as a lipid selenium binding drug, can regulate metabolic disorders through multiple pathways and play a target organ protective role in a variety of diseases. Its main active ingredient is Gypenoside A. Numerous studies have shown that Gypenoside A regulate lipid metabolism, improve cardiovascular function, and exhibit anti-fibrotic, anti-inflammatory, and anti-tumor properties^32^-^34^. Clinically, they are used to treat hyperlipidemia and atherosclerosis, among other cardiovascular diseases. Studies have shown that Gypenoside A can treat myocardial ischemia in rabbits induced by coronary artery ligation, alleviate coronary artery spasms, arrhythmias, and elevated blood pressure caused by posterior pituitary hormones, and improve cardiac ejection function^35^-^37^. Research on cerebrovascular injury also demonstrated that Gypenoside A protect brain tissue by inhibiting NO overexpression, reducing acute focal cerebral ischemia-reperfusion injury and oxidative stress in rats, and increasing antioxidant enzyme activity^38^. Based on previous studies, we hypothesize that Gypenoside A can protect against vascular endothelial injury associated with AS. Although prior studies have confirmed the endothelial protective and anti-oxidative effects of Gypenoside A, the downstream pathological mechanisms and therapeutic targets remain unclear^39^.

This study aims to further elucidate the regulatory role of mitophagy and necroptosis phenotypes in the pathological mechanisms of vascular endothelial injury associated with atherosclerosis through animal and cell experiments. In doing so, we seek to identify potential therapeutic targets and promising pharmacological agents while clarifying the pharmacological effects and regulatory mechanisms of anti-AS drugs like Gypenoside A.

## Methods

### Animals

All procedures and animal experiments strictly adhered to the US National Institutes of Health Guidelines for the Care and Use of Laboratory Animals. These were further endorsed by the Guangzhou University of Traditional Chinese Medicine. Approval for all experimental procedures related to the treatment and surgery of the animal model was granted by the Animal Care and Use Committees of the University of Guangzhou university of traditional chinese medicine^40^. The mice were maintained on a 12-hour light/dark cycle with free access to food and water. Male ApoE knockout mice (C57BL/6) were given a high-cholesterol diet (HCD) for 4 weeks to induce an early atherosclerosis model. Mice in different groups were administered varying doses of Gypenoside A (as a pretreatment at levels of 5 mg/kg and 10 mg/kg/day) and mitophagy activators/inhibitors (FCCP and 3MA 30 mg/kg) for 8 weeks; Lipid metabolism markers and inflammation levels were assessed using the respective assay kits. Vascular injury and the expression of markers associated with injury pathways were evaluated through tissue immunofluorescence staining. Simultaneously, targeted and untargeted metabolomics were utilized to analyze differential metabolite expression in animals subjected to varying dose gradients and genetic modifications. TMBIM6/NDUFS-4 gene-deficient mouse models were established based on previous research to investigate the role of TMBIM6/NDUFS-4 regulation in the development of AS.

### Metabolomics testing

The GC-MS analysis workflow involved metabolite preprocessing, extraction, derivatization, GC-MS detection, data processing^41^, and statistical analysis for each group of mice^42^. Non-targeted metabolomics based on quadrupole mass spectrometry, combined with the MS-DIAL software for metabolomics data analysis, was applied to perform qualitative and relative quantitative analysis of the raw data, followed by standardized preprocessing. The differences in metabolomics data are illustrated in the figures and tables presented in the article^43^.

### Immunofluorescence

After sample collection, arterial tissue specimens were immediately preserved at -20ºC or -80ºC under optimal cutting temperature conditions. Frozen tissue samples were sectioned into 4µm-thick pathological tissue sections^44^. Immunofluorescence staining was conducted on tissue sections that were fixed for 15 minutes in 10% formalin under low-temperature conditions^45^. The sections were washed three times with PBST and incubated with 10% goat serum at room temperature for 1 hour. The primary antibody (Caspase-9) was subsequently applied, and the sections were incubated overnight at 4ºC. After three subsequent washes, the sections were stained using the corresponding fluorescently conjugated secondary antibody. Nuclear staining was performed using DAPI, and imaging of these sections was conducted with a Zeiss LSM 510 microscope^46^, ^47^.

### Mitophagy assay

Mito-Keima is a fluorescent protein marker that serves as an indicator of Mitophagy activation. According to previous research, in cells expressing Mito-Keima, mitophagic activity was evaluated by measuring the ratio of cells with high 561/457 nm fluorescence (red/green), which indicates Mitophagy activation^47^.

### Cell culture and treatment

HUVECs were obtained from the Experimental Center of Guangzhou University of Traditional Chinese Medicine. Ox-LDL was purchased from Shanghai Jingke Chemical Technology Co., Ltd. HUVECs were routinely cultured in RPMI 1640 medium and preconditioned with ox-LDL (100 mg/L) for 24 hours to establish a lipid damage model^48^. Cells from different groups were pretreated with Gypenoside A(a concentration of 60 ug/ml for 48 h), mitophagy inhibitors, or activators (FCCP/UA, Urolithin A, 5 mM) based on the specific modeling and intervention conditions. According to previous studies, gene silencing and overexpression of target genes (TMBIM6/NDUFS4/VDAC1) were performed using cell transfection techniques for subsequent cellular experiments^49^.

### CCK8 and ELISA

Cell viability was assessed using the CCK8 assay following the protocol provided by the manufacturer^50^. Inflammatory cytokines, caspase family proteins, and oxidative stress markers (IL-17, IL-10, TNF-α, Caspase-3, Caspase-9, Caspase-12, SOD, MDA, GPX) were quantified using ELISA kits (Nanjing Jiancheng Bioengineering Institute, Nanjing, China), following the manufacturer's instructions. The levels of relevant markers in serum or cell supernatants were used for analysis^51^.

### qPCR

Total RNA was extracted from frozen pathological tissue or cell specimens with the RNeasy Mini Kit (Qiagen), and cDNA was synthesized through reverse transcription^52^. We used SYBR Green Master Mix (Roche) to conduct real-time quantitative PCR under a stringent 40-cycle thermal protocol on multiple genes and targets. Gene expression levels in cardiomyocytes from different groups were quantified using the 2^-ΔΔCt^ method. Details of the primers used in our PCR study are provided in the supplementary table^53^.

### Statistical analysis

Data were presented as mean ± standard deviation (SD). Statistical analyses were performed using SPSS 19.0 and GraphPad Prism 9.0. A two-tailed Student's t-test was used for comparisons between two groups, and one-way ANOVA for multiple group comparisons. Bonferroni post-hoc tests were applied for subsequent analyses if the assumption of homogeneity of variance was met; Otherwise, Tamhane's T2 test was used for heterogeneity of variance. A p-value below 0.05 was deemed statistically significant.

## Results

### Mitochondrial Pathway-Mediated Apoptosis as a Potential Key Mechanism in the Development of Atherosclerosis

**Figure 1:**
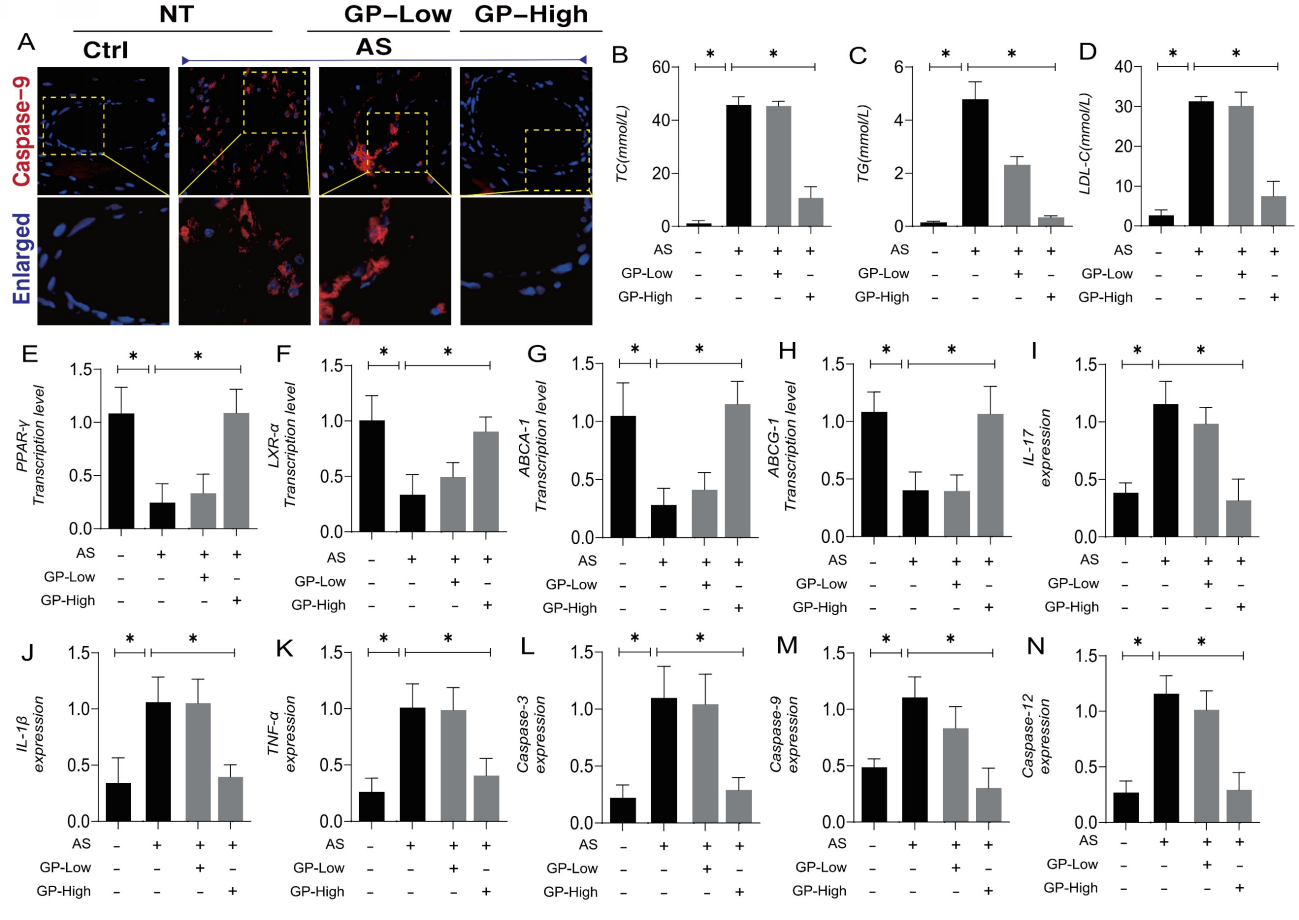
** Gypenoside (GP)regulates atherosclerotic vascular injury through mitochondrial pathway. (A)** The expression level of caspase-9 was detected by immunofluorescence; **(B-D)** Total cholesterol (TC), triglyceride (TG), low-density lipoprotein cholesterol (LDL-C) levels of mice in each group (n = 10); **(E-H)** The expression of cholesterol reverse transport (RCT) - related genes (PPAR γ, LXR α, ABCA1 and ABCG1) was detected by Q-PCR;**(I-K)** ELISA was used to detect the expression level of inflammatory factors IL-17 / IL-1β / TNF-α; **(L-N)** The expression levels of Caspase-3, caspase-9 and caspase-12 in mitochondrial pathway were detected by ELISA; Values are presented as mean ± SD. All experiments were conducted in triplicate. *p < 0.05.

Atherosclerosis (AS) is a chronic, progressive vascular inflammation, marked by the accumulation of lipids within the arterial walls and a concomitant inflammatory response. Numerous studies have demonstrated that inflammation plays a key role in the formation and progression of cardiovascular and cerebrovascular diseases associated with AS. Beyond inflammation, pathological factors such as lipid accumulation, oxidative stress, and mitochondrial metabolic dysregulation interact, further complicating the process of lesion formation. To explore potential therapeutic agents targeting AS-induced stress damage, as well as the interaction mechanisms involving lipotoxicity and vascular injury-associated metabolic disorders, we established an AS animal model and conducted interventions using different dosage gradients of Gypenoside A (GP). Immunofluorescence results from vascular tissues revealed overexpression of Caspase-9 following model establishment, alongside elevated expression of TC, TG, and LDL-C (Figure 1A-D). These findings suggest that AS is accompanied by the activation of the Caspase-9 pathway and abnormalities in lipid metabolism.

Further experimental studies showed that, as AS pathology progressed, mice exhibited suppressed transcription levels of PPAR-γ, LXR-α, ABCA-1, and ABCG-1, indicating that AS is associated with downregulated expression of lipid metabolism regulatory genes (Figure 1E-H). Studies related to the inflammatory storm in atherosclerosis also indicated overexpression of inflammatory cytokines IL-17, IL-1β, and TNF-α, along with elevated expression of Caspase-3, Caspase-9, and Caspase-12(Figure 1I-N). These results further suggest that vascular damage associated with AS may lead to the activation of inflammatory storms and mitochondrial pathway-mediated programmed cell death.

Pharmacological experiments revealed that high-dose Gypenoside A intervention could reverse these phenomena, reducing the overexpression of lipid metabolites and increasing the transcription levels of lipid metabolism regulatory genes (Figure 1A-H). Additional experimental results also indicated that high-dose Gypenoside A can suppress the overexpression of mitochondrial pathway apoptosis regulatory factors, such as Caspase-3, Caspase-9, and Caspase-12, and inhibit the overexpression of inflammatory cytokines (Figure 1I-N). Immunofluorescence results further demonstrated that high doses of Gypenoside A could inhibit the overexpression of Caspase-9 surrounding vascular tissues (Figure 1A). The findings from animal experiments suggest that Gypenoside A may serve as a promising therapeutic agent for AS, with its regulation of mitochondrial pathway-mediated programmed cell death and lipid metabolism likely serving as critical therapeutic targets for the treatment of AS.

### Metabolomic Validation of the Key Mechanisms by Which Gynostemma Pentaphyllum Total Saponins Improve Atherosclerosis

**Figure 2:**
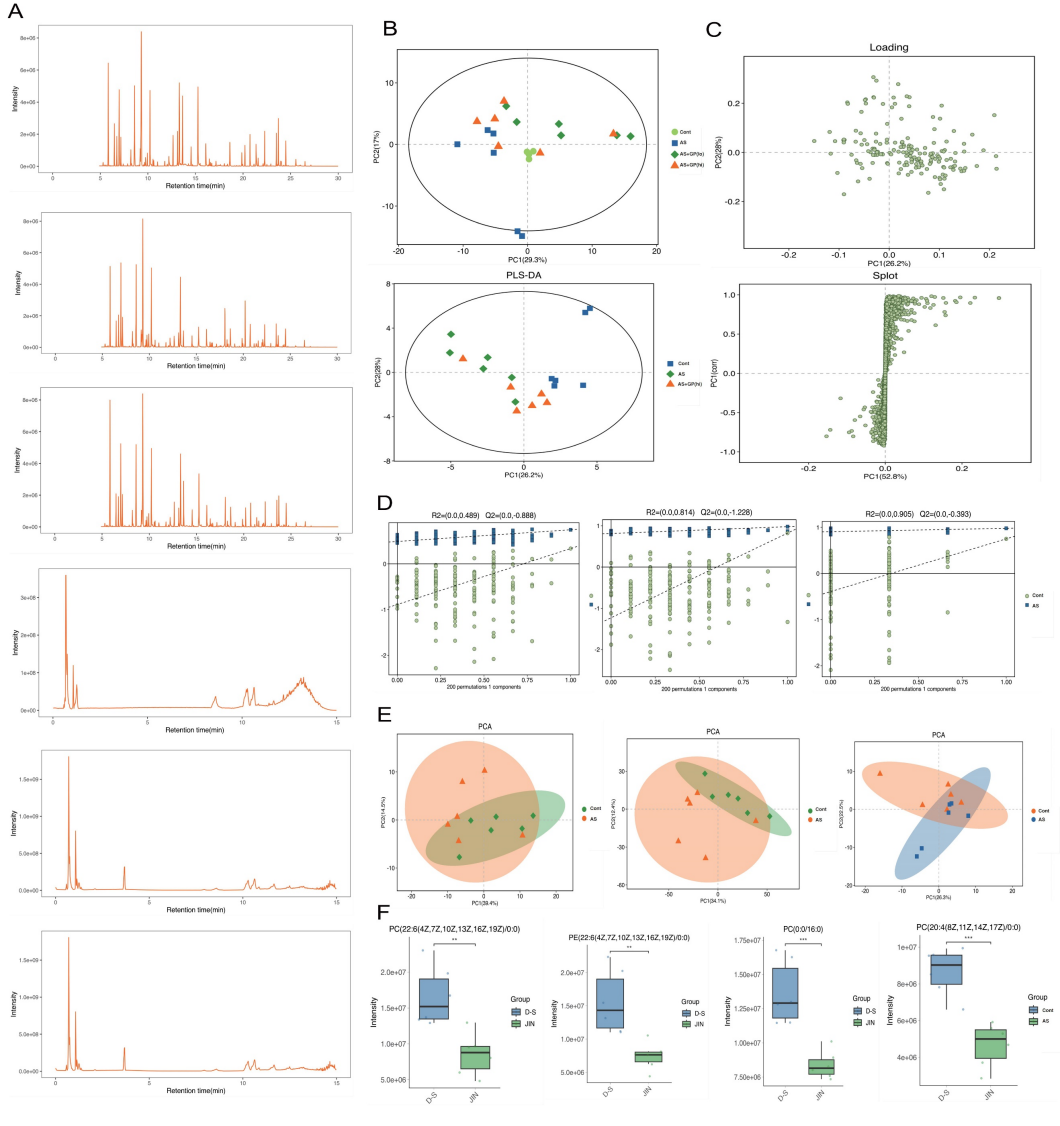
** Metabolomic study of Gypenoside regulating atherosclerotic vascular injury via mitochondrial pathway (mass spectrometry and quality control). (A)** Base peak chromatogram; **(B)** Principal component analysis of samples; **(C)** Pls Da loading diagram; **(D)** Permutation plot; **(E)** PCA plot; **(F)** Box diagram of differential metabolites; Values are presented as mean ± SD. All experiments were conducted in triplicate. *p < 0.05.

**Figure 3:**
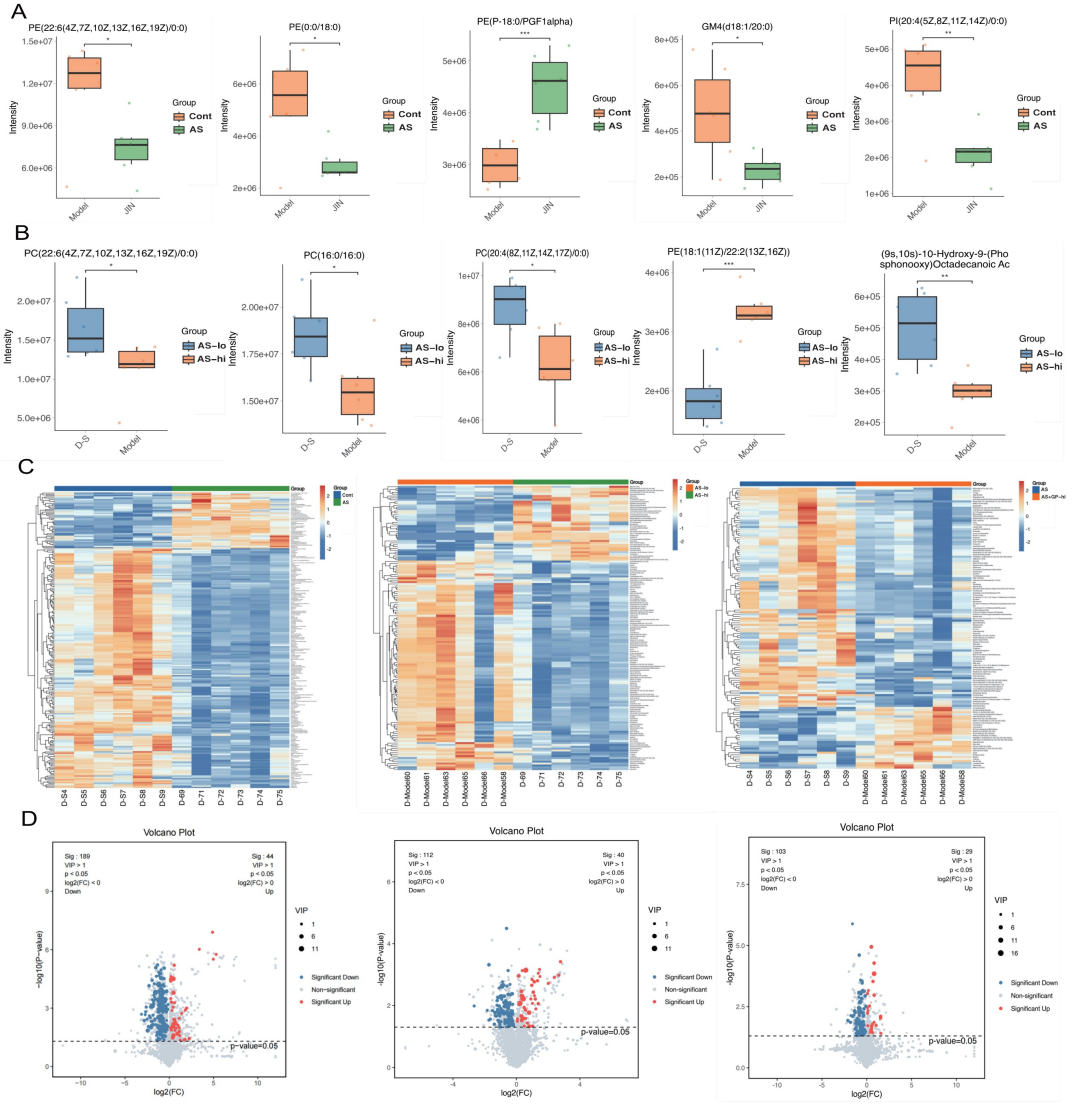
** Metabonomic study on Gypenoside regulating atherosclerotic vascular injury through mitochondrial pathway (difference comparative analysis). (A-B)** boxplot of differential metabolites; **(C)** Heatmap of differential metabolite clustering; **(D)** Volcano plot of differential metabolites; Values are presented as mean ± SD. All experiments were conducted in triplicate. *p < 0.05.

**Figure 4:**
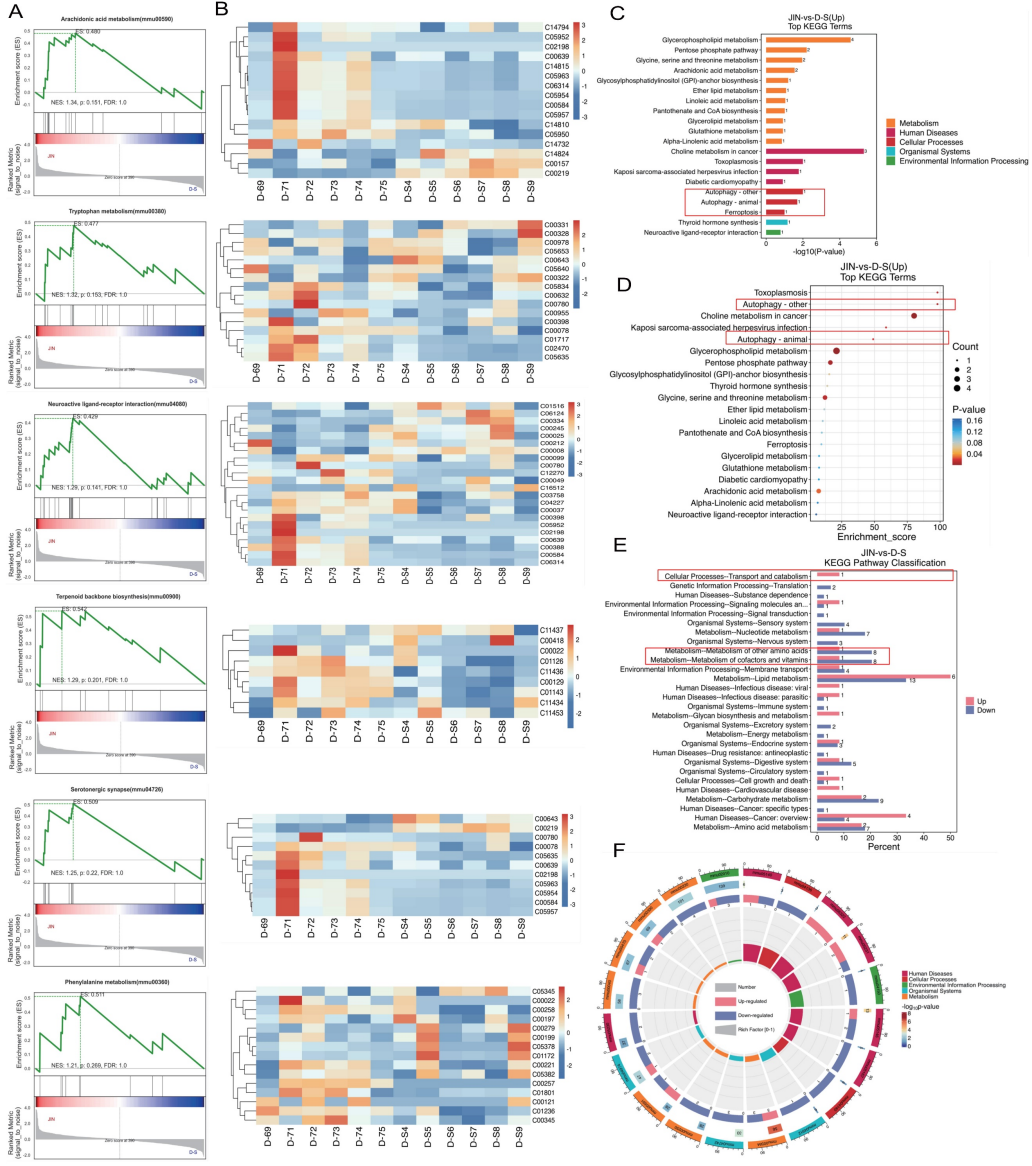
** Metabonomic study of Gypenoside regulating atherosclerotic vascular injury via mitochondrial pathway (cluster gene target screening). (A)** GSEA enrichment analysis; **(B)** GSEA clustering diagram; **(C-E)** KEGG level3 level distribution map of differential metabolites; **(F)** KEGG enrichment analysis circle diagram; Values are presented as mean ± SD. All experiments were conducted in triplicate. *p < 0.05.

To further elucidate the pharmacological mechanisms and therapeutic targets of Gypenoside A (GD) in anti-atherosclerosis (AS), we conducted a metabolomic study. This study uniquely employed a combination of UPLC-QTOF and HPLC-MS/MS metabolomic methods to analyze and detect serum metabolite levels. The results indicated that the AS model had a significant impact on serum metabolites in mice, and GD treatment partially reversed these effects (Figure 2A-F). To further explore whether changes in circulating metabolites in AS mice could be key phenotypes involved in the pathological mechanisms, we performed histograms and volcano plot analyses (Figure 3A-D). The findings revealed substantial variations in metabolite levels, with intergroup differences being highly significant. Further experiments revealed that the enriched differential metabolites and genes were closely related to autophagy, metabolism, and programmed cell death (Figure 4A-F).

**Figure 5:**
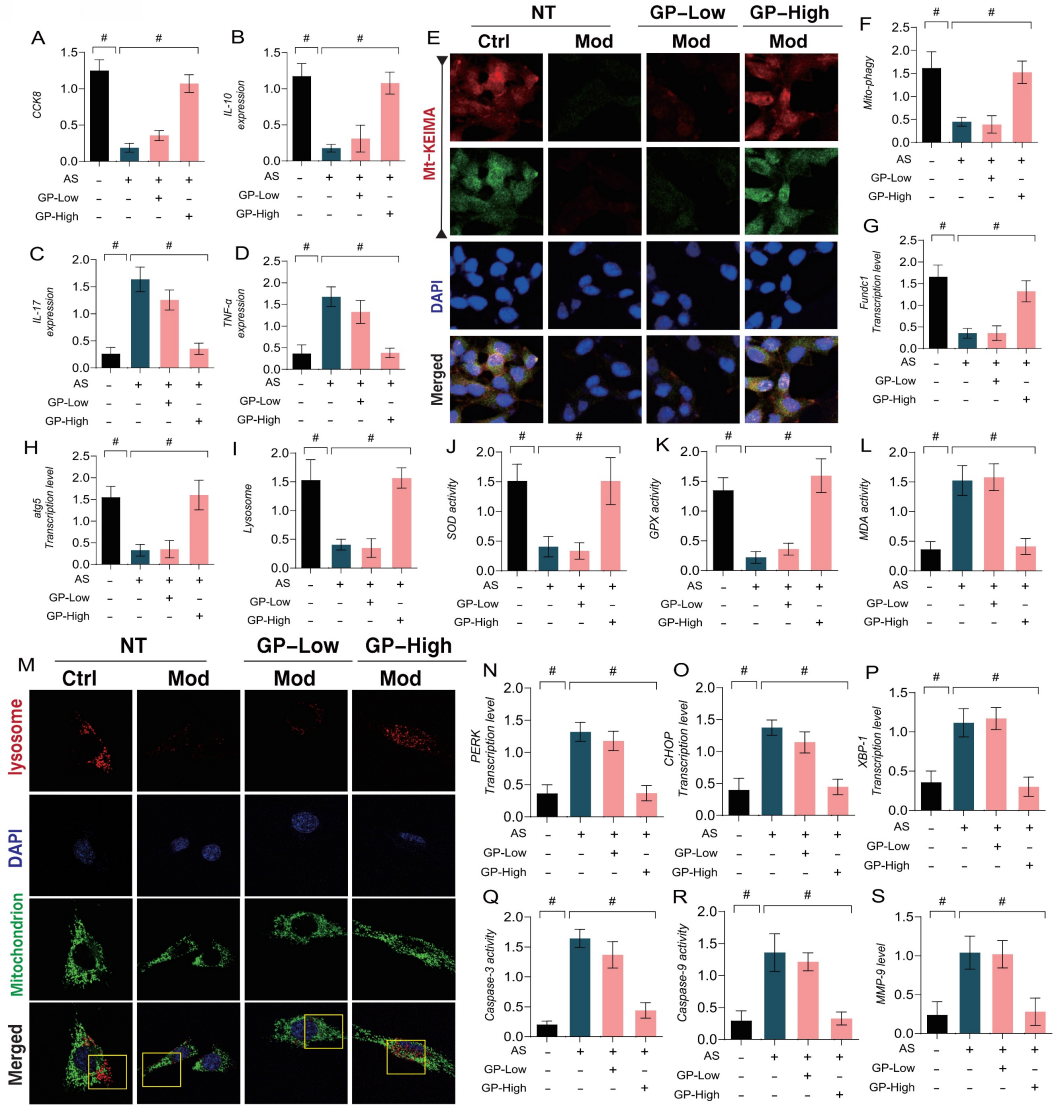
** GP regulates oxidative stress and inflammatory damage in vascular endothelial cells through Mitophagy endoplasmic reticulum stress. (A)** CCK8 detection of cell activity; **(B-D)** ELISA was used to detect the activity of anti-inflammatory factor IL-10 and inflammatory factor IL-17/TNF-α; **(E-F)** mt KEIMA was used to detect the expression level of Mitophagy; **(G)** The transcription level of FUNDC1;**(H)** The transcription level of ATG5; **(I, M)** Immunofluorescence expression levels of Mitophagy lysosomes (confocal detection); **(J)** The expression level of SOD;**(K)** The expression level of GPX;**(L)** The expression level of MDA;**(N)** The transcription level of PERK; **(O)** The transcription level of CHOP; **(P)** The transcription level of XBP-1; **(Q)** Caspase-3 activity; **(R)** Caspase-9 activity; **(S)** The expression level of MMP-9;Values are presented as mean ± SD. All experiments were conducted in triplicate. *p < 0.05.

In addition, we explored the mechanisms of GD in regulating vascular endothelial damage associated with AS by using a lipid toxicity model of endothelial cells. Ox-LDL was applied to induce endothelial damage, followed by CCK8 assays to assess cell viability. The results indicated a marked reduction in cell viability in the model group when compared to the control group, accompanied by a reduction in anti-inflammatory factors and an increase in inflammatory markers (Figure 5A-D). Immuno-fluorescence and qPCR further confirmed that mitophagy marker levels were significantly reduced in endothelial cells after modeling, along with a marked decrease in the transcription levels of FUNDC1 and ATG5(Figure 5E-H). These results suggest that lipid toxicity may lead to increased levels of inflammatory factors, coupled with reduced mitophagy levels, which could be a major factor contributing to decreased endothelial cell viability. Notably, high doses of GD intervention reversed these effects, enhancing mitophagy and anti-inflammatory factor activity while inhibiting inflammatory factor expression (Figure 5A-H). However, low doses of GD did not exhibit significant therapeutic effects (Figure 5A-H). These findings align with the metabolomic results, suggesting that autophagy and mitophagy are key regulatory mechanisms mediating endothelial damage following AS.

Furthermore, our study investigated the regulatory mechanisms of oxidative stress and ER stress in endothelial damage following AS. The results indicated that lipid-induced endothelial cell damage led to a significant decrease in mitochondrial-lysosome expression, accompanied by overexpression of oxidative stress markers (MDA) and a reduction in antioxidant enzyme activity (Figure 5I-S). Further experiments confirmed that the transcription levels of ER stress marker genes PERK, CHOP, and XBP-1 were significantly elevated, along with increased expression of mitochondrial pathway-related inflammatory apoptosis regulators (Caspase-3, Caspase-9, and MMP-9) (Figure 5I-S). However, high-dose GD intervention reversed these phenomena, reducing the transcription levels of ER stress markers and the expression of inflammatory apoptosis regulators, while activating mitophagy and enhancing antioxidant enzyme activity. In contrast, low-dose GD did not regulate the activation of mitophagy, ER stress, or oxidative stress pathways (Figure 5 I -S).

### Gypenoside A Regulate “Mitochondria-ER” Homeostasis via the NDUFS4-TMBIM6-VDAC1 Axis to Improve Endothelial Cell Damage

**Figure 6:**
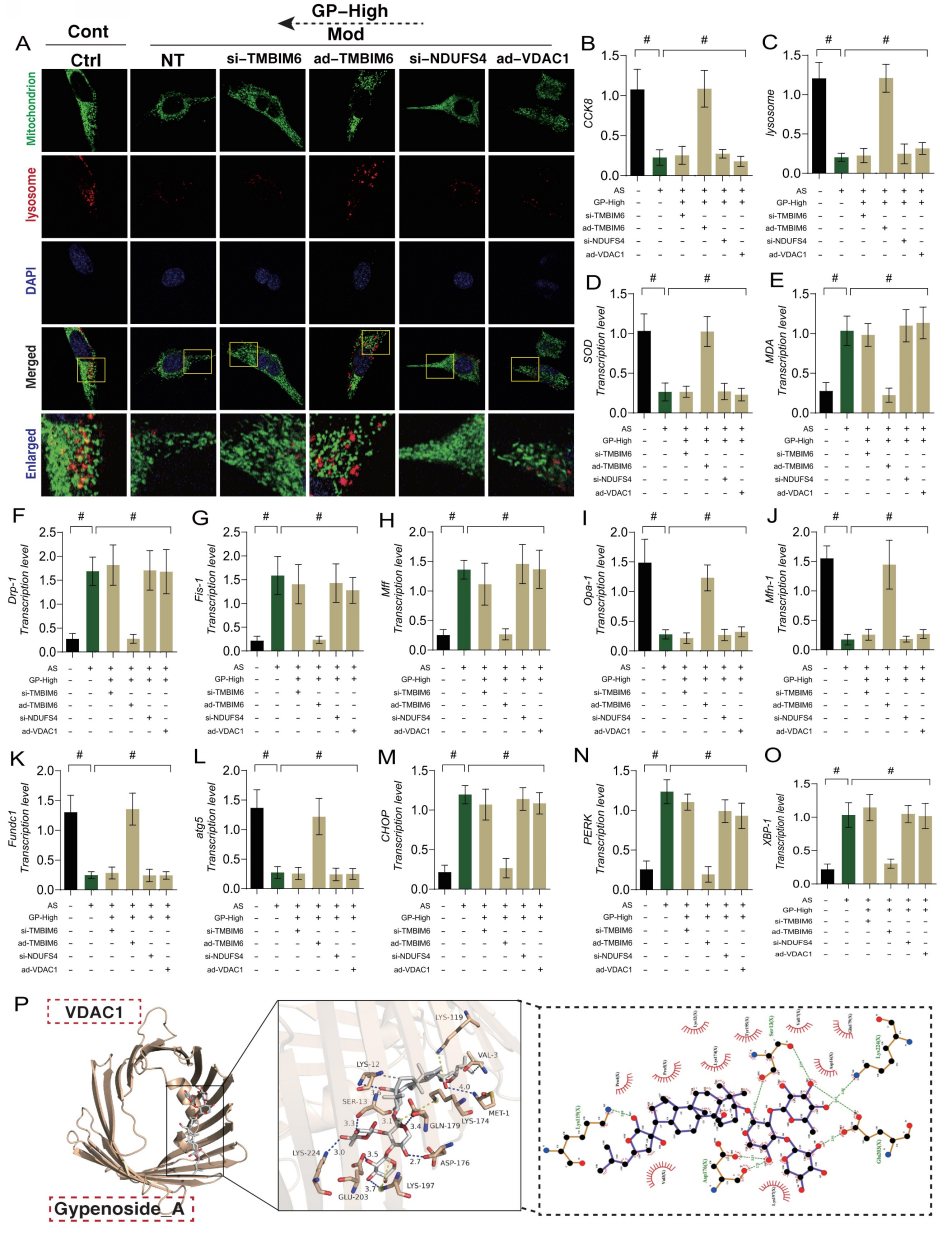
** GP improves vascular endothelial cell damage through TMBIM6-VDAC1 mediated Mitophagy mitochondrial dynamics. (A, C)** Expression levels of Mitophagy lysosomes (laser confocal microscopy);**(B)** CCK8 detection of cell activity;**(D)** The expression level of SOD;**(E)** The expression level of MDA;**(F)** The transcription level of Drp-1;**(G)** The transcription level of Fis-1;**(H)** The transcription level of Mff;**(I)** The transcription level of Opa-1;**(J)** The transcription level of Mfn-1;**(K)** The transcription level of FUNDC1;**(L)** The transcription level of Atg5;**(M)** The transcription level of CHOP;**(N)** The transcription level of PERK;**(O)** The transcription level of XBP-1; **(P)** Molecular docking prediction(VDAC1 and GP); Values are presented as mean ± SD. All experiments were conducted in triplicate. *p < 0.05.

ER plays a critical role in regulating phenotypic abnormalities in protein folding, transport, and post-translational modifications in endothelial cells following AS. It also participates in the synthesis and metabolism of lipids and sterols. Under conditions of mitochondrial dysfunction and cellular homeostasis disruption, the ER is also affected, leading to the accumulation of misfolded or unfolded proteins in the ER lumen. When ER stress exceeds its capacity, it triggers a pathological mechanism of ER stress dysfunction, which is closely related to mitophagy and mitochondrial dynamics dysfunction. To verify the impact of GD on endothelial cell injury and its upstream regulatory mechanisms, we genetically modified endothelial cells by knocking down or overexpressing TMBIM6, NDUFS4, and VDAC1. Molecular docking prediction experiment found that VDAC1 and GP could produce a high binding force (-8.8) (Figure 6 P), suggesting that GP could produce protein regulatory effect on VDAC1.Subsequent molecular biology experiments examined mitochondrial-lysosome expression, mitophagy, mitochondrial dynamics (fusion and fission), and ER stress-regulating genes. The results showed that cell viability decreased with reduced mitophagy levels, alongside elevated mitochondrial oxidative stress and increased mitochondrial fission, while mitochondrial fusion decreased (Figure 6 A-O). Significant ER stress was also observed (Figure 6 M-O). GD intervention reversed these phenomena.

Notably, the therapeutic effects of GD were abolished when TMBIM6 and NDUFS4 were knocked down, or when VDAC1 was overexpressed. These results further confirmed that the regulatory effects of GD on mitophagy, mitochondrial dynamics, and ER stress are likely mediated through the NDUFS4-TMBIM6-VDAC1 axis (Figure 6 A-O). However, the specific mechanisms require further experimental validation. These findings are consistent with our previous research, where we demonstrated that Ginsenoside Rb1 regulates mitochondrial dynamics in heart failure mice, primarily through the TMBIM6-VDAC1 axis. In myocardial inflammation, the NLRP3-mediated inflammatory response was also found to interact with mitochondrial quality control, exacerbating myocardial damage under stress conditions. Ginsenoside Rb1 was shown to regulate the DUSP-1-TMBIM6-VDAC1 axis, inhibiting the release of pro-inflammatory factors and altering the metabolic composition in heart failure mice, protecting the damaged myocardium, consistent with the results of this study.

### Gypenoside A Regulate Endothelial Cell Necroptosis via the NDUFS4-TMBIM6-VDAC1 Axis to Improve Endothelial Cell Damage

**Figure 7:**
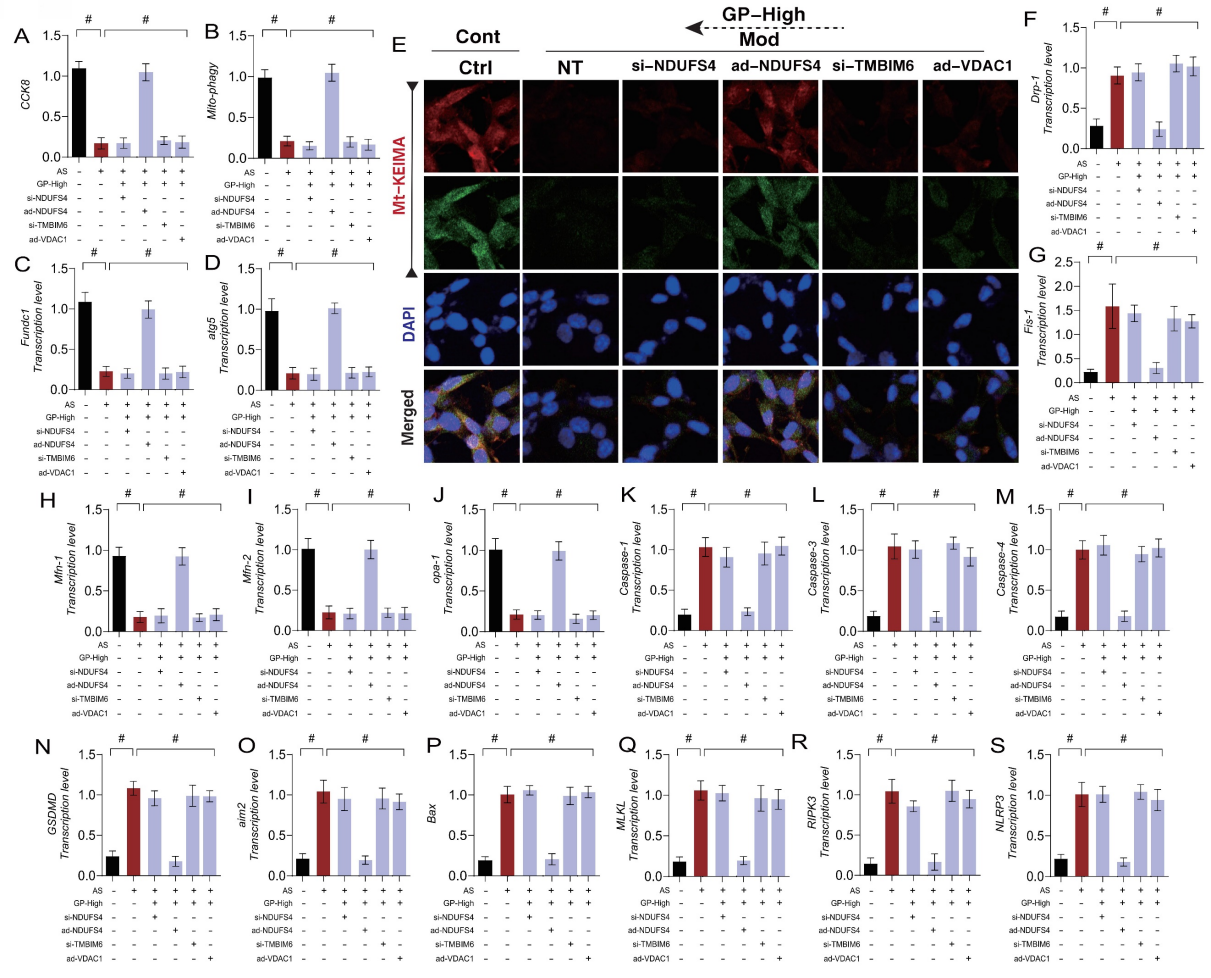
** GP improves vascular endothelial cell damage through NDUFS-4-TMBIM6-VDAC1 axis mediated cell necrosis apoptosis. (A)** CCK8 detection of cell activity;**(B, E)** Mt-Keima detection of Mitophagy expression levels;**(C)** The transcription level of FUNDC1;**(D)** The transcription level of Atg5;**(F)** The transcription level of Drp-1;**(G)** The transcription level of Fis-1;**(H)** The transcription level of Mfn-1;**(I)** The transcription level of Mfn-2;**(J)** The transcription level of Opa-1;**(K)** The transcription level of Caspase-1;**(L)** The transcription level of Caspase-3;**(M)** The transcription level of Caspase-4;**(N)** The transcription level of GSDMD;**(O)** The transcription level of AIM2;**(P)** The transcription level of Bax;**(Q)** The transcription level of MLKL;**(R)** The transcription level of RIPK3;**(S)** The transcription level of NLRP3;Values are presented as mean ± SD. All experiments were conducted in triplicate. *p < 0.05.

Cell death is a normal physiological process, and different mechanisms of cell death may play distinct roles in disease processes. In the pathogenesis of AS, we identified mitochondrial pathway-mediated necroptosis in endothelial cells, but its specific mechanism remains unclear. To clarify how GD regulates mitophagy and mitochondrial pathway-mediated necroptosis through the NDUFS4-TMBIM6-VDAC1 axis, we established a gene-modified cell experimental system targeting NDUFS4, TMBIM6, and VDAC1. The results demonstrated that GD reversed mitophagy dysfunction and mitochondrial dynamics imbalance, inhibiting the transcription levels of mitochondrial pathway necroptosis-related genes (Figure 7 A-S). The findings suggest that AS modeling leads to mitophagy dysfunction and mitochondrial dynamics imbalance, which in turn activates mitochondrial pathway necroptosis.

Interestingly, knocking out NDUFS4 and TMBIM6 or overexpressing VDAC1 reversed the effects of GD, consistent with previous results (Figure 7 A-S). However, overexpression of NDUFS4 did not affect GD's regulatory role in mitophagy, mitochondrial dynamics, or mitochondrial pathway necroptosis (Figure 7 A-S). These results suggest that GD improves endothelial cell mitochondrial homeostasis and reverses mitochondrial pathway necroptosis by regulating the NDUFS4-TMBIM6-VDAC1 axis. Based on these findings, we hypothesize that NDUFS4-TMBIM6-VDAC1-mediated mitophagy is a key regulatory pathway for GD in modulating mitochondrial pathway necroptosis in endothelial cells and represents a potential therapeutic target.

### Gypenoside A Improve Endothelial Cell Damage by Regulating Mitophagy Homeostasis via NDUFS4

**Figure 8:**
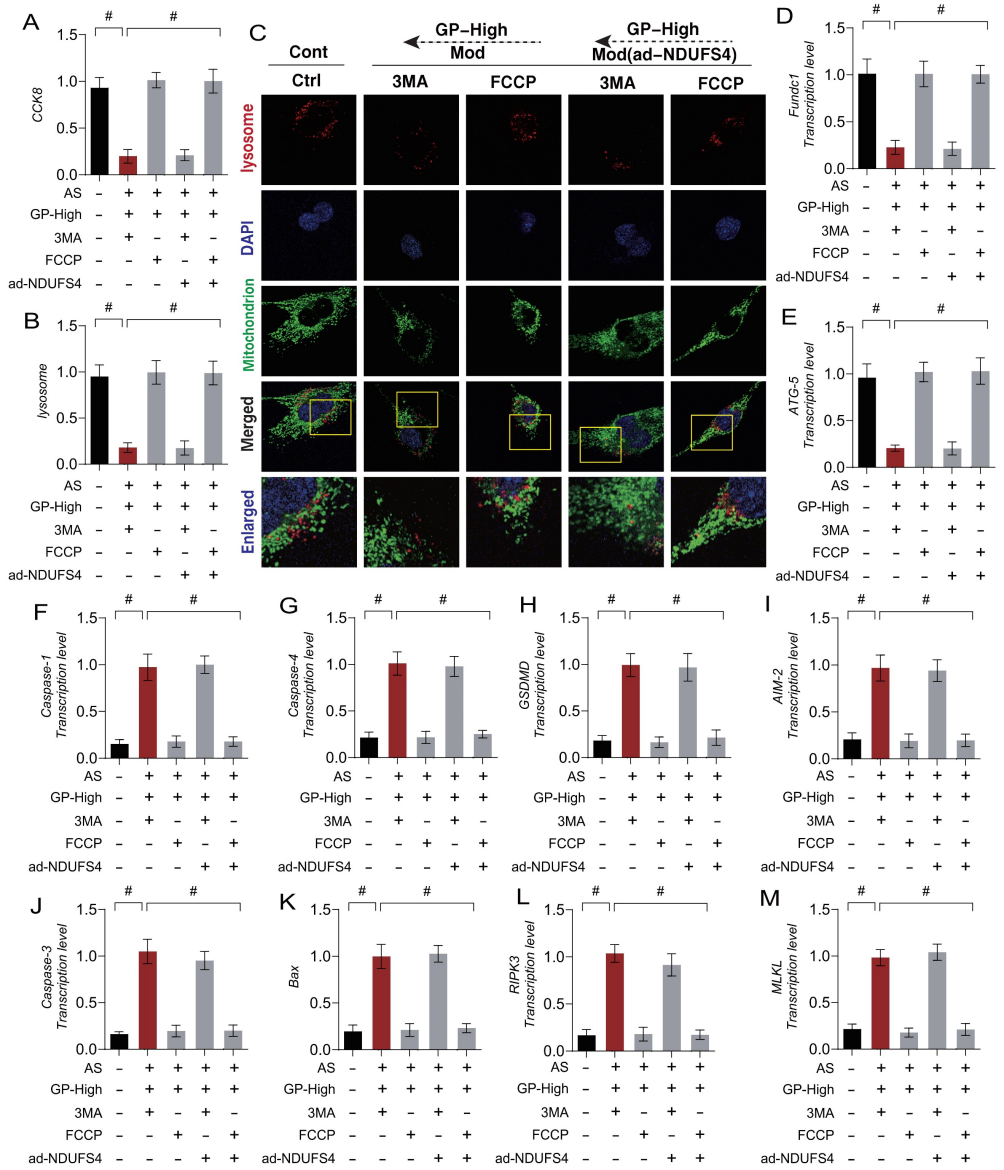
** GP regulates cellular necroptosis through NDUFS-4 mediated Mitophagy pathway. (A)** CCK8 detection of cell activity; **(B, C)** Expression levels of Mitophagy lysosomes (laser confocal microscopy); **(D)** The transcription level of FUNDC1; **(E)** The transcription level of Atg5; **(F)** The transcription level of Caspase-1; **(G)** The transcription level of Caspase-4; **(H)** The transcription level of GSDMD;**(I)** The transcription level of AIM2; **(J)** The transcription level of Caspase-3; **(K)** The transcription level of Bax; **(L)** The transcription level of MLKL; **(M)** The transcription level of RIPK3;Values are presented as mean ± SD. All experiments were conducted in triplicate. *p < 0.05.

To investigate the mechanism by which Gypenoside A (GD) regulate mitophagy through NDUFS4 to improve endothelial cell damage, we applied autophagy inhibitors (3MA) and autophagy activators (FCCP) in combination with GD to intervene in AS-mimicking endothelial cell injury. The experimental results showed that the autophagy inhibitor (3MA) abolished the regulatory effects of GD on mitophagy and mitochondrial pathway necroptosis, while the mitophagy activator (FCCP) did not affect GD's regulatory effects (Figure 8 A-M).

These findings suggest a close connection between GD's modulation of mitochondrial pathway necroptosis in endothelial cells and mitophagy. To further confirm the role of NDUFS4 in GD's regulation of mitophagy in endothelial cells, we performed overexpression of NDUFS4 (ad-NDUFS4) while applying autophagy inhibitors (3MA) and activators (FCCP) (Figure 8 A-M). The results indicated that ad-NDUFS4 treatment did not affect GD's regulatory effects on mitophagy and necroptosis, further supporting the critical role of NDUFS4 in this mechanism.

### Gypenoside A Mediate Mitophagy-Dynamics Regulation of ER Stress and Necroptosis via the NDUFS4-TMBIM6 Axis

**Figure 9:**
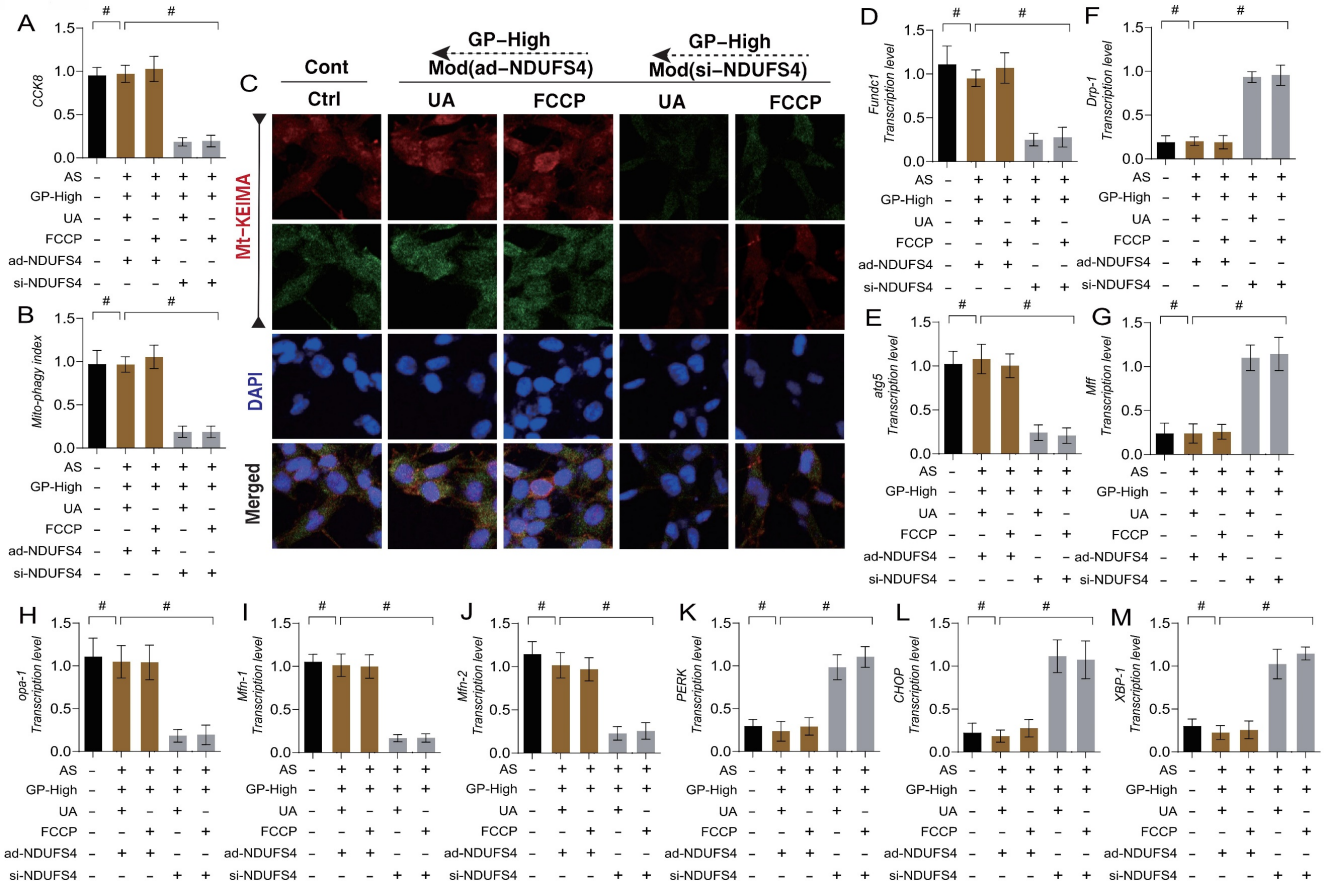
** GP regulates endothelial cell necroptosis through the mitochondrial dynamics endoplasmic reticulum stress pathway via NDUFS-4. (A)** CCK8 detection of cell activity; **(B, C)** Mt-Keima detection of Mitophagy expression levels; **(D)** The transcription level of FUNDC1; **(E)** The transcription level of Atg5; **(F)** The transcription level of Drp-1; **(G)** The transcription level of Mff; **(H)** The transcription level of Opa-1; **(I)** The transcription level of Mfn-1; **(J)** The transcription level of Mfn-2; **(K)** The transcription level of PERK; **(L)** The transcription level of CHOP; **(M)** The transcription level of XBP-1;Values are presented as mean ± SD. All experiments were conducted in triplicate. *p < 0.05.

To verify the mechanism by which GD regulates ER stress and necroptosis in endothelial cells through NDUFS4-mediated mitophagy, we again applied mitophagy activators (UA/FCCP) in combination with GD to intervene in AS-mimicking endothelial cell injury. The experimental results showed that mitophagy activation with UA/FCCP did not effects on ER stress and mitochondrial pathway necroptosis (Figure 9 A-M). To further clarify the role of NDUFS4 in GD's regulation of mitophagy, we knocked down NDUFS4 (si-NDUFS4) while applying mitophagy activators (UA/FCCP). The results showed that si-NDUFS4 treatment abolished the regulatory effects of GD and mitophagy activators (UA/FCCP) on mitophagy, ER stress, and mitochondrial pathway necroptosis. These findings suggest that GD likely improves endothelial injury by regulating mitophagy-ER stress via NDUFS4, which in turn modulates endothelial cell necroptosis (Figure 9 A-M).

**Figure 10:**
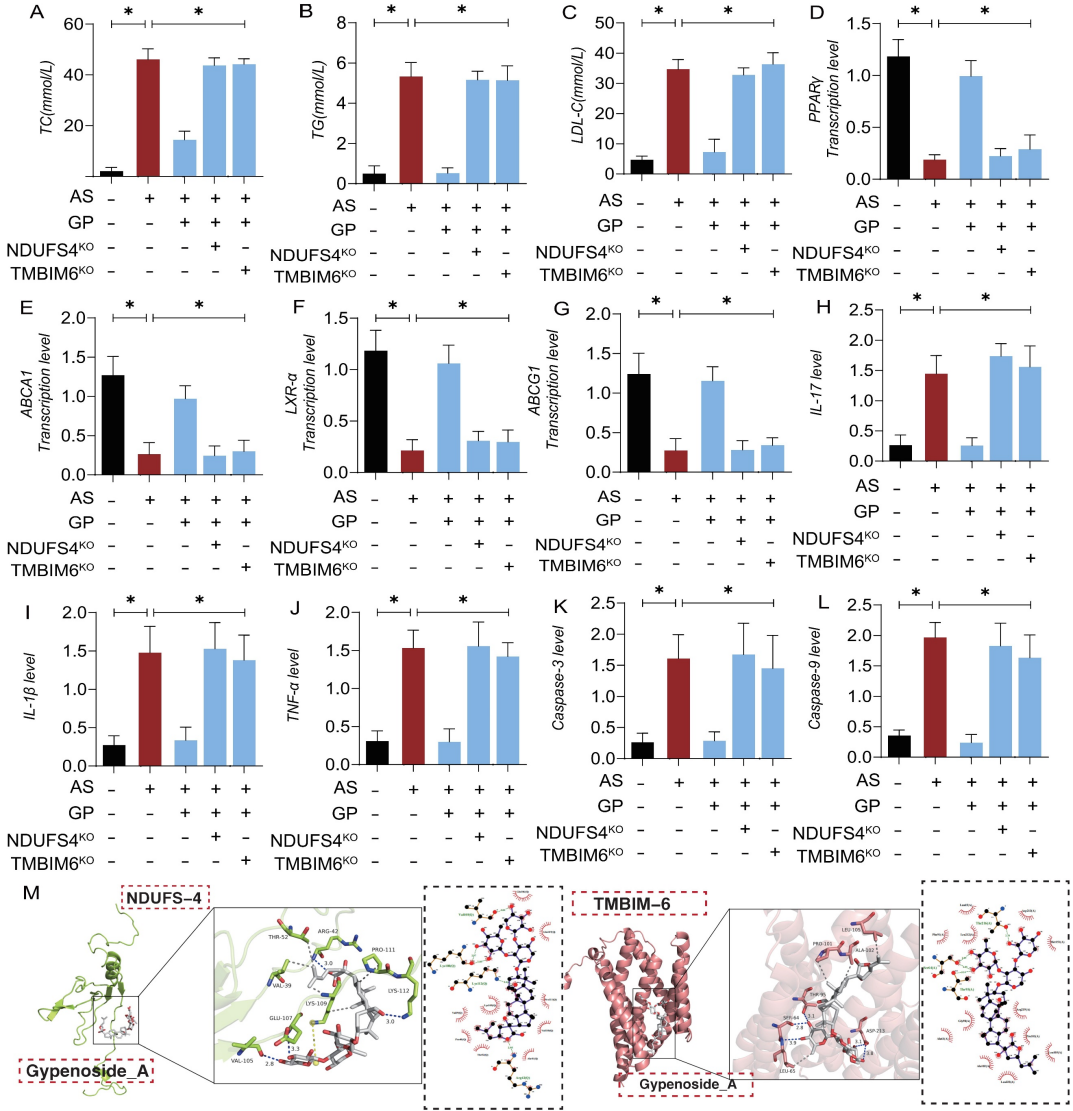
** Gypenosides regulate atherosclerotic vascular injury through NDUFS4. (A-C)** Total cholesterol (TC), triglycerides (TG), and low-density lipoprotein cholesterol (LDL-C) levels in each group of mice (n=10); **(D-G)** q-PCR detection of the expression of cholesterol reverse transport (RCT) related genes (PPAR γ, LXR α, ABCA1, and ABCG1); **(H-J)** ELISA was used to detect the expression levels of inflammatory factors IL17/IL-1β/TNF-α; **(K-L)** ELISA was used to detect the expression levels of apoptosis factors Caspase-3 and Caspase-9 in mitochondrial pathway cells;** (M)** Molecular docking prediction(TMBIM6/NDUFS4 and GP);Values are presented as mean ± SD. All experiments were conducted in triplicate. *p < 0.05.

In animal experiments, we further demonstrated that GD regulates lipid metabolism and mitochondrial pathway inflammatory damage/necrosis through the NDUFS4-TMBIM6 axis. Molecular docking prediction experiment found that NDUFS4-TMBIM6 and GP can produce high binding force (Figure 10 M), suggesting that GP can produce protein regulatory effect on NDUFS4-TMBIM6. The experimental results showed that high-dose GP modulated the expression of lipid metabolites and the transcription levels of lipid metabolism-regulating genes, subsequently regulating the expression of pro-inflammatory and anti-inflammatory factors. Additionally, it activated the expression of mitochondrial pathway necroptosis-related regulatory genes (Figure 10 A-L). However, NDUFS4-TMBIM6 gene deletion abolished the regulatory effects of GP on lipid metabolism, mitochondrial pathway inflammatory damage, and necroptosis (Figure 10 A-L). This is consistent with the experimental results of molecular docking, and these results are consistent with previous cell experiments, suggesting that GP regulates lipid metabolism and vascular endothelial inflammation after AS through the NDUFS4-TMBIM6 axis, thereby preventing endothelial cell programmed death. The experimental results of NDUFS4-TMBIM6-related gene modified mice further suggested that Gypenoside A has strong advantages in regulating lipid metabolism, vascular environment homeostasis.

## Discussion

This study utilized animal and cellular gene modification techniques, along with metabolomic analysis, to further validate the role of TMBIM6-NDUFS4-mediated mitophagy in the pathological mechanisms of atherosclerosis (AS). Additionally, it revealed the complex interactions between pathological mitochondrial fission, inflammatory storms, and oxidative stress following vascular endothelial injury. These findings confirmed the mechanisms by which therapeutic drugs regulate mitophagy phenotypes and upstream targets, providing critical insights for the development of AS therapies and vascular-protective drugs. The key discoveries of this research are as follows: (1) AS activates an inflammatory storm, leading to endothelial inflammation and oxidative stress, and further lead to metabolic disorders; (2) Pharmacological interventions (GD) can mitigate lipid toxicity-induced endothelial dysfunction, reduce inflammatory damage, and inhibit mitochondrial pathway-mediated necroptosis; It can also further regulate the metabolic disorder and intestinal flora abundance disorder in post as model mice. (3) The gene knockout of TMBIM6-NDUFS4 reverses the regulatory effects of drugs on endothelial cells and lipid metabolism, resulting in mitochondrial pathway-induced endothelial injury; and there may be a significant interaction between TMBIM6 and NDUFS4 that directly mediates mitophagy. Collectively, these findings highlight potential therapeutic targets for AS and lipid-induced vascular endothelial damage^54^.

TMBIM6, also referred to as BAX inhibitor-1 (BI-1), is an anti-apoptotic factor that plays a vital role in preventing apoptosis and necroptosis in cardiomyocytes, vascular endothelial cells, and various organ tissues^55^. Its anti-apoptotic function primarily prevents the activation of BAX and its translocation to mitochondrial sites^56^. Additionally, research has found that TMBIM6 is also localized on the surface of the endoplasmic reticulum (ER), where it regulates mitochondrial and ER homeostasis and promotes the release of Ca²⁺ from the ER to mitochondria^57^, thereby normalizing ER function and calcium homeostasis^56^, ^58^. Previous experimental results demonstrated that the loss of TMBIM6 leads to calcium release dysregulation in mice, accompanied by impaired mitophagy, reduced myocardial oxygen consumption rates, and ATP synthesis defects. Prior studies also showed that after exposure to lipopolysaccharides (LPS), the expression levels of myocardial TMBIM6 in wild-type mice were significantly suppressed, resulting in cardiac ejection dysfunction and an inflammatory storm in the myocardium and vascular endothelium. These pathological damages were further exacerbated in TMBIM6 cardiac-specific knockout mice. Furthermore, we found that the loss of TMBIM6 increased mitochondrial susceptibility to LPS in cardiomyocytes, leading to impaired mitochondrial metabolic function and exacerbated oxidative stress. These results suggest that the loss of TMBIM6 may be a critical regulatory mechanism influencing LPS-mediated inflammatory storms and mitochondrial fission/fusion imbalances^51^.

In another experimental study, we discovered that a mitochondrial-protective traditional Chinese medicine (ZSHX) exhibited dose-dependent efficacy in alleviating ischemic myocardial injury by enhancing mitochondrial integrity. The cardioprotective and mitochondrial-preserving effects of ZSHX were impaired by *TMBIM6* knockout (*TMBIM6^CKO^*), while the benefits of ZSHX persisted in TMBIM6 transgenic (*TMBIM6^TG^*) mice^19^. Furthermore, *TMBIM6^CKO^* inhibited the mitochondrial function of cardiomyocytes treated with ZSHX. Hypoxia disrupted mitochondrial quality control (MQS) in cardiomyocytes, leading to calcium overload, excessive fission, impaired autophagy, and biosynthesis interruptions. ZSHX mitigated these effects, restoring mitochondrial quality control (MQS), reducing calcium overload, and preventing cardiomyocyte necrosis^59^. Additionally, our findings demonstrated that hypoxia-induced inhibition of TMBIM6 led to excessive activation of VDAC1, a critical mitochondrial channel. ZSHX enhanced TMBIM6 expression and suppressed the VDAC1-mediated calcium uptake pathway, thereby alleviating calcium overload and improving MQS function^46^. Another research result reveal TMBIM6 as a key regulator of tubular function, maintaining mitochondrial localization of PHB2 and highlighting new therapeutic prospects for combating AKI^58^. TMBIM6 has been identified as a sentinel and executor of AS-mediated vascular endothelial damage, leading to vascular injury caused by mitochondrial homeostasis dysregulation in endothelial cells^60^. Therefore, targeting the regulation of TMBIM6 may represent a key therapeutic pathway for the treatment of AS in the future. Ndufs4 is a crucial regulatory protein involved in maintaining intracellular homeostasis. Previous studies have shown that it plays an important role in Mitophagy, mitochondrial energy metabolism, mitochondrial oxidative stress, and the regulation of macrophage polarization^61^.

Additionally, Ndufs4 has been found to coordinate the delicate balance between macrophage and osteoclast polarization. As a fundamental subunit of mitochondrial complex I (CI), Ndufs4 acts as a modulator of innate immunity and skeletal homeostasis, thereby regulating the macrophage-osteoclast lineage transition. The absence of Ndufs4 suppresses osteoclast differentiation and bone resorption, while promoting macrophage activation and inflammation through both cell-autonomous and systemic mechanisms^62^. Further research has revealed that respiratory complex I (NADH: ubiquinone oxidoreductase) is essential for cellular energy metabolism and NAD^+^ homeostasis mechanisms. Cryo-electron microscopy analysis of complex I in *ndufs4^-/-^* mouse hearts demonstrated a loose association between the NADH dehydrogenase module and a discrete region containing the assembly factor NDUFAF2 or the subunit NDUFS6^63^, ^64^. In mature complex I, the subunit NDUFA12 replaces its homolog NDUFAF2, but this substitution is absent across all categories, exacerbating the effects of NDUFS4 deficiency and preventing the maturation of the enzyme lacking NDUFS4^65^. The loss of ndufs4 triggers a cascade of pathological mechanisms associated with mitochondrial diseases, providing us with new insights into these processes at the molecular level^64^. Building on previous research, this study further confirms the mechanism by which NDUFS4 influences AS-mediated vascular endothelial injury^66^. The experimental results suggest that the drug primarily exerts its effects through NDUFS4, and the loss of NDUFS4 exacerbates the inflammatory storm and oxidative stress damage induced by AS. This process is mediated by Mitophagy dysfunction and the activation of pathological mitochondrial fission, leading to increased necroptosis through the mitochondrial pathway. Additionally, the results indicate that NDUFS4 interacts with TMBIM6, which helps maintain mitochondrial homeostasis and normalizes Mitophagy, thus modulating mitochondrial-related programmed cell death pathways.

Extensive evidence highlights the role of VDAC1 in vascular injury-related diseases^67^-^69^. The experiment employed siRNA to knock down VDAC1 or inhibited its upstream regulators by targeting TMBIM6^70^. VDAC1-mediated cardiomyocyte injury and mitochondrial energy metabolism dysfunction have been identified as key mechanisms driving necroptosis, a process thought to be mediated by TMBIM6^71^. When TMBIM6 expression is low, hypoxia-induced stress in cardiomyocytes is associated with blocked Mitophagy, disrupted mitochondrial dynamics, excessive pathological fission, subsequent activation of RIPK3 and MLKL, mitochondrial morphological and structural damage, and significantly reduced cardiomyocyte viability^46^, ^72^, ^73^. However, pharmacological intervention promoted TMBIM6 overexpression, inhibited VDAC1 phosphorylation, activated FUNDC1 phosphorylation at Tyr18, normalized Mitophagy, and restored cardiomyocyte viability^74^. These findings are consistent with our experimental results, which confirmed *in vitro* and *in vivo* that the drug modulates VDAC1 through the TMBIM6-NDUFS4 axis, maintaining normal Mitophagy and inhibiting mitochondrial fission, thereby blocking mitochondrial pathway-mediated inflammatory storms and oxidative stress damage while protecting vascular endothelial cells. The TMBIM6-NDUFS4-VDAC1 axis may represent a crucial therapeutic target for regulating AS-induced endothelial and plaque damage^75^.

In summary, these findings position the TMBIM6-NDUFS4-VDAC1 axis at the center of AS-mediated endothelial and plaque inflammatory damage. Given the critical role of the TMBIM6-NDUFS4-VDAC1 interaction in mitochondrial homeostasis regulation, exploring potential therapeutics targeting this axis could offer promising treatments for metabolic disorder-related endothelial damage. Mitophagy has already been shown to play a key role in the early stages of atherosclerosis, where endothelial cells exhibit a significant increase in mitochondrial ROS, accumulation of mtDNA damage, and progressive respiratory chain dysfunction, resulting in endothelial cell (EC) dysfunction and VSMC phenotype switching^76^, ^77^. The activation of Mitophagy can remove these mutated or damaged mitochondria, preserving mitochondrial homeostasis and maintaining the regulatory mechanisms of mitochondrial energy metabolism^78^. Additionally, studies have shown that Mitophagy exerts a “dual” regulatory effect on cardiac function, where excessive autophagy can trigger programmed cell death in endothelial cells, leading to endothelial damage and an inflammatory storm^79^, ^80^.

Although our study preliminarily confirmed the role of the TMBIM6-NDUFS4-VDAC1 axis in regulating AS injury and endothelial inflammation/oxidative stress damage, several limitations remain: (1) In the animal experiments, we did not further confirm the protein and transcriptional expression levels of TMBIM6 and NDUFS4, and only established AS models through gene modification experiments. Future research should include molecular biology assessments and mitochondrial protein expression analyses to validate TMBIM6 and NDUFS4 expression at the animal level. (2) In the cellular experiments, we only confirmed the targeted regulatory roles of Mitophagy and mitochondrial dynamics in AS pathology but did not explore the roles of mitochondrial biogenesis and the unfolded protein response in maintaining intracellular homeostasis and mitigating inflammatory storms. We plan to further investigate these regulatory mechanisms through molecular biology experiments in the future. (3) At the molecular biology level, while we confirmed the role of the TMBIM6-NDUFS4-VDAC1 regulatory axis in mitochondrial pathway-mediated endothelial cell programmed death, we did not elucidate the interaction mechanism of the TMBIM6-NDUFS4-VDAC1 axis through co-immunoprecipitation. Future experiments will focus on protein interaction studies to further clarify the role of TMBIM6-NDUFS4-VDAC1 in AS pathogenesis.
